# Emergency Physician-performed Transesophageal Echocardiography in Simulated Cardiac Arrest

**DOI:** 10.5811/westjem.2017.5.33543

**Published:** 2017-07-19

**Authors:** Don V. Byars, Jordan Tozer, John M. Joyce, Michael J. Vitto, Lindsay Taylor, Turan Kayagil, Matt Jones, Matthew Bishop, Barry Knapp, David Evans

**Affiliations:** * Eastern Virginia Medical School, Department of Emergency Medicine, Norfolk, Virginia; † Virginia Commonwealth University, Department of Emergency Medicine, Richmond, Virginia

## Abstract

**Introduction:**

Transesophageal echocardiography (TEE) is a well-established method of evaluating cardiac pathology. It has many advantages over transthoracic echocardiography (TTE), including the ability to image the heart during active cardiopulmonary resuscitation. This prospective simulation study aims to evaluate the ability of emergency medicine (EM) residents to learn TEE image acquisition techniques and demonstrate those techniques to identify common pathologic causes of cardiac arrest.

**Methods:**

This was a prospective educational cohort study with 40 EM residents from two participating academic medical centers who underwent an educational model and testing protocol. All participants were tested across six cases, including two normals, pericardial tamponade, acute myocardial infarction (MI), ventricular fibrillation (VF), and asystole presented in random order. Primary endpoints were correct identification of the cardiac pathology, if any, and time to sonographic diagnosis. Calculated endpoints included sensitivity, specificity, and positive and negative predictive values for emergency physician (EP)-performed TEE. We calculated a kappa statistic to determine the degree of inter-rater reliability.

**Results:**

Forty EM residents completed both the educational module and testing protocol. This resulted in a total of 80 normal TEE studies and 160 pathologic TEE studies. Our calculations for the ability to diagnose life-threatening cardiac pathology by EPs in a high-fidelity TEE simulation resulted in a sensitivity of 98%, specificity of 99%, positive likelihood ratio of 78.0, and negative likelihood ratio of 0.025. The average time to diagnose each objective structured clinical examination case was as follows: normal A in 35 seconds, normal B in 31 seconds, asystole in 13 seconds, tamponade in 14 seconds, acute MI in 22 seconds, and VF in 12 seconds. Inter-rater reliability between participants was extremely high, resulting in a kappa coefficient across all cases of 0.95.

**Conclusion:**

EM residents can rapidly perform TEE studies in a simulated cardiac arrest environment with a high degree of precision and accuracy. Performance of TEE studies on human patients in cardiac arrest is the next logical step to determine if our simulation data hold true in clinical practice.

## INTRODUCTION

Emergency physicians (EP) routinely use transthoracic echocardiography (TTE) in the evaluation of critically ill patients, including those in cardiac arrest, to aid in diagnosis and guide therapy. Despite the diagnostic value of TTE, it is frequently limited by patient habitus, ongoing cardiopulmonary resuscitation (CPR) efforts, mechanical ventilation, and interference from monitoring equipment. Transesophageal echocardiography (TEE) is an established and accurate method of evaluating heart anatomy and function that, due to its indwelling location, is not affected by the common limitations associated with TTE. These characteristics have shown to be beneficial in cardiac arrest; by helping to identify causes and guiding CPR efforts.^1^–^4^ TEE has many potential advantages over TTE for the patient in cardiac arrest, including the ability to image the heart in real time during active CPR. Furthermore, TEE has a well-established safety profile in the elective setting.^5^ Yet despite these advantages, EPs have been slow to implement TEE in their practice.^6^–^7^

The ability to learn TEE skills on simulators has been demonstrated in a several specialties.^8^–^13^ However, prior studies have not examined the ability of emergency medicine (EM) residents to acquire and retain TEE skills, nor have they demonstrated the ability of trainees to identify pathologies commonly seen during cardiac arrest. Thus, the following study was designed as a prospective, simulation-based study that aimed to evaluate the ability of EM residents to perform TEE. During the study, participants had to learn and retain TEE image-acquisition techniques and demonstrate those skills to diagnose common pathological conditions during simulation on a high-fidelity TEE model.

## METHODS

This was a multicenter trial in which 40 EM resident physicians took part in a didactic- and simulation-based educational initiative that took place in four consecutive weekly sessions. Each session was 30 minutes in length and took place at the simulation center of each institution. An ultrasound (US) faculty member with a Registered Diagnostic Cardiac Sonographer certification and TEE experience taught each session. The institutional review boards of both institutions approved the study protocol.

Residents who were able to complete all four sessions were identified for inclusion. All residents had training in basic emergency bedside US and standard TTE imaging, a two-day introductory US course at a minimum, but they had varying levels of cardiac experience, as residents from all three post-graduate years of training were included. None had any prior TEE experience.

The first session included a 15-minute didactic lecture given by an EP with experience in TEE and outlined information on transesophageal US, including transducer manipulation, image acquisition, and emergency applications. Participants were taught a quick look two-view protocol including both the mid-esophageal four-chamber (ME4C) and the mid-esophageal two-chamber (ME2C) views (Figure).

Population Health Research Capsule

What do we already know about this issue?

Transesophageal echocardiography is a technique that may provide superior diagnostic capabilities in cardiac arrest, but its use is limited.

What was the research question?

Can emergency medicine residents learn limited TEE views and diagnose common cardiac arrest pathologies in simulation?

What was the major finding of the study?

EM residents can rapidly perform TEE studies in a simulated cardiac arrest environment with high degree of precision and accuracy.

How does this improve population health?

Dissemination of this technique may facilitate further studies into its effect on cardiac arrest outcomes.

After the didactic lecture, each resident was given instruction and a tutorial of the high-fidelity simulator and TEE probe (Vimedix, CAE Inc). Each participant was then instructed how to obtain both the mid-esophageal four- and two-chamber views and allowed to learn the controls of the TEE probe. Comparison anatomy to TTE was provided to the participants to aid in knowledge retention.

The study participants were then brought back for a second and third session in two subsequent consecutive weeks, and engaged in proctored image acquisition of pre-determined pathology. Each participant was required to insert the probe, obtain each of the required two views and then name the pathology to the instructor. This process was repeated with multiple pathologies, including cardiac tamponade, asystole, acute myocardial infarction MI (severely diminished ejection fraction, regional wall motion abnormalities), fine ventricular fibrillation (VF), and normal images.

Assessment was performed during the fourth and final session. Each participant was individually tested to determine his/her ability to quickly perform the required views and make the critical diagnosis. They were instructed to insert the probe and obtain a four-chamber view, a two-chamber view and identify the pathology if any was present. Time started from the beginning of probe insertion and concluded after all the above criteria were met. Probe insertion time was a minimal component to the overall elapsed time of the procedure. No questions or clarification were allowed, and no help in obtaining the images was provided. Additionally, access to the simulator was restricted to the practice and testing sessions only, with no additional training provided.

We entered data directly into a study-specific spreadsheet. The spreadsheet recorded time to diagnosis, quality of images, and correct diagnosis. We summarized descriptive statistics using means and standard deviations (SD). Inter-rater reliability was estimated using a kappa (k) statistics, with k = 0.61–0.80 interpreted as “good agreement” and k > 0.80 interpreted as “very good agreement.”

## RESULTS

Forty EM residents from two different academic medical centers completed four consecutive weekly sessions, three for training and one for testing. They represented all three post-graduate years of EM training (Table 1).

After three consecutive weekly education sessions, testing was performed. During testing, six simulated cardiac arrest cases were presented in random order: asystole, cardiac tamponade, VF, acute MI, and two normal cases.

For each simulated cardiac arrest case, subjects were evaluated on the time to obtain both ME4C and ME2C views and the ability to correctly diagnose pathology. This resulted in 80 normal TEE studies and 160 pathologic TEE studies, for 240 total studies. In all cases, the subjects were able to insert the TEE probe into the simulator and successfully obtain both views, resulting in 100% success rate for both.

Our calculations for the ability to diagnose the cardiac pathology encountered in this simulation study by EM residents resulted in a cumulative sensitivity of 98% (95% confidence interval [CI] [95–99%]), specificity of 99% (96%–100%), positive likelihood ratio of 78.0 (11.1–547.1), and negative likelihood ratio 0.025 (0.009–0.067) (Table 2).

The sensitivity per pathology was as follows: asystole 100% (95% CI, [100–100%]), tamponade 98% (93–100%), VF 98% (93–100%), acute MI 95% (88–100%). The average time to diagnose each objective structured clinical examination case was normal in 31 sec ± 15 (SD); asystole in 11 ± 5.5; tamponade in 14 sec ± 8; acute MI in 21 sec ± 10; and VF in 12 sec ± 4.4**.** This included time for probe insertion, time to obtain both views, and time to make the interpretation. Inter-rater reliability between EPs was extremely high, resulting in a k coefficient across all cases of 0.95.

## DISCUSSION

TEE is a well-established diagnostic modality whose usefulness is now being explored by EPs in the care of critically ill patients and those in cardiac arrest. It has the potential to eliminate many of the barriers commonly associated with TTE in that setting, while providing higher quality diagnostic images and simultaneously allowing external interventions such as CPR. As such, there has been a push towards further dissemination of these skills to more EPs.^6^,^9^ The use of TEE simulators has recently been demonstrated to be an effective method of training in multiple fields including EM, cardiology and cardiac anesthesia, all for users without prior exposure to TEE.^8^–^13^ In contrast to prior studies that have focused on attending- and fellow-level learners, this study demonstrated that after a series of brief training sessions, EM residents can easily and routinely obtain two TEE views, the mid-esophageal four chamber and mid-esophageal two chamber. Furthermore, this study has shown that they can identify four pathologic conditions causing cardiac arrest in a simulated environment with a high degree of sensitivity and specificity.

All study participants were successful in obtaining both mid-esophageal four- and two-chamber views. These views were selected because they are easy to obtain and require little manipulation of the probe. They also provide images that are easily comparable to TTE images, thus allowing quick recognition of structures and pathology. The high success rate is likely because of the minimal probe manipulation required to obtain these views and is in keeping with a prior TEE simulation study.^9^ In contrast to Arntfield et al., we did not ask participants to obtain more technically difficult gastric views, as these require more probe manipulation and time, and would be unlikely to provide any further discriminating information in the setting of real or simulated cardiac arrest. This is supported by an observational review, which noted that TEE had diagnostic influence in 78% of cases, during which a ME4 view was obtained 96% of the time, with all other views to a uniformly lesser degree.^6^

There were study participants from all three post-graduate years who completed the full study protocol, with varying levels of experience in echocardiography. Despite that, after just three brief training sessions, they were able to easily obtain two routine TEE views and identify common pathologic conditions in cardiac arrest with great success and high inter-rater reliability. This is the first study to evaluate the ability of EM residents to perform TEE, and shows that it can be easily taught and retained in the simulated setting. While this study should be repeated in the live patient, it may help to disprove one barrier to the more widespread practice of this modality, which is that limited two-view TEE is a difficult skill to learn.

## LIMITATIONS

One of the limitations of this study is that it was a simulation-based training and testing protocol. Despite the use of a high-fidelity simulator, the study is subject to the limitations inherent within this paradigm. Specifically, in this study participants were asked to accurately diagnose pathologies during the testing phase that they had previously seen during training. On the simulator, this means the identical cases or images were used. While pattern recognition plays an important role in the practice of medicine, the simulator is not able to present the variability that would be seen in real-world cases, which may therefore result in an upward skew of the sensitivity and specificity.

Another limitation involves the translation of haptic skills between probe insertion and manipulation on a simulator model versus the live patient. It is inherently easier on the simulator, which may result in increased success rate with obtaining the required views of this protocol. Lastly, the EM residents who completed this voluntary study may be more experienced, motivated learners than those who did not complete all the study sessions. This may have resulted in increased sensitivity and specificity, and perhaps less generalizability to the general resident population.

## CONCLUSION

After a series of brief teaching sessions, EM residents with varying levels of experience in echocardiography were able to uniformly obtain two standard TEE views and diagnose common pathologic conditions in simulated cardiac arrest with a high degree of sensitivity, specificity, and inter-rater reliability. This is the first study to evaluate the diagnostic abilities of physicians using TEE in a simulated cardiac arrest setting, and the first to evaluate the ability of EM residents to learn TEE skills. Further research efforts are needed to determine if the success of this study can be repeated in the in-vivo setting, and if the diagnostic benefits translate to improvements in survival.

## Figures and Tables

**Figure ab:**
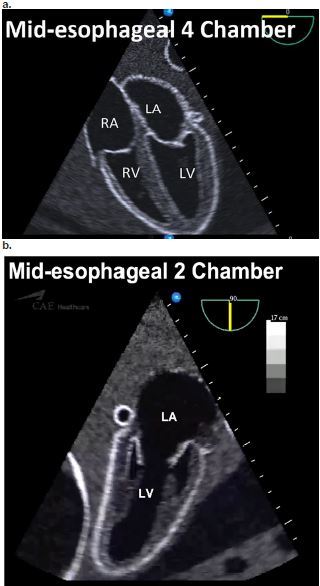
Simulator images of the quick look two-view protocol for transesophageal echocardiography *RA,* right atrium; *LA*, left atrium; *RV*, right ventricle; *LV*, left ventricle.

**Table 1:** Training year and institution distribution of emergency medicine residents who participated in a transesophageal echocardiography simulation study.

	Virginia Commonwealth University	Eastern Virginia Medical School
PGY1	6	6
PGY2	5	8
PGY3	7	8

*PGY,* post-graduate year.

**Table 2:** Cumulative sensitivity, specificity, positive and negative likelihood ratios for transesophageal echocardiography during simulated cardiac arrests, across all pathology.

	Sensitivity (95% CI)	Specificity (95% CI)	LR+ (95% CI)	LR− (95% CI)
All cases	0.98 (0.95–0.99)	0.99 (0.96–1.00)	78.0 (11.1–547.1)	0.025 (0.009–0.067)

*CI*, confidence interval.
